# Towards Long-Term Stable Polyimide-Based Flexible Electrical Insulation for Chronically Implanted Neural Electrodes

**DOI:** 10.3390/mi12111279

**Published:** 2021-10-20

**Authors:** Andreas Schander, Julia M. Gancz, Marcel Tintelott, Walter Lang

**Affiliations:** 1 Institute for Microsensors, -Actuators and -Systems (IMSAS), University of Bremen, 28359 Bremen, Germany; juliamgancz@gmail.com (J.M.G.); tintelott@iwe1.rwth-aachen.de (M.T.); wlang@imsas.uni-bremen.de (W.L.); 2 Institute of Materials in Electrical Engineering 1, RWTH Aachen University, 52074 Aachen, Germany

**Keywords:** polyimide, neural interfaces, flexible implants, long-term stability, interdigital electrode array, electrical insulation stability, chronic implants

## Abstract

For chronic applications of flexible neural implants, e.g., intracortical probes, the flexible substrate material has to encapsulate the electrical conductors with a long-term stability against the saline environment of the neural tissue. The biocompatible polymer polyimide is often used for this purpose. Due to its chemical inertness, the adhesion between two polyimide layers is, however, a challenge, which can lead to delamination and, finally, to short circuits. The state-of-the-art method to improve the adhesion strength is activating the polyimide surface using oxygen reactive ion etching (O_2_ RIE). However, the influence of the process variations (etching time, bias power) on the long-term stability is still unclear. Therefore, we establish a test method, where the aging of a gold interdigital structure embedded in two polyimide layers and immersed in saline solution is accelerated using an elevated temperature, mechanical stress and an electrical field. A continuous measurement of a leakage current is used to define the failure state. The results show that the variation of the O_2_ RIE plasma process has a significant effect on the long-term stability of the test samples. Comparing the two different plasma treatments 0.5 min at 25 W and 1 min at 50 W, the long-term stability could be increased from 20.9 ± 19.1 days to 44.9 ± 18.9 days. This corresponds to more than a doubled lifetime. An ideal solution for the delamination problem is still not available; however, the study shows that the fine-tuning of the fabrication processes can improve the long-term stability of chronically implanted neural electrodes.

## 1. Introduction

**Figure 1:**
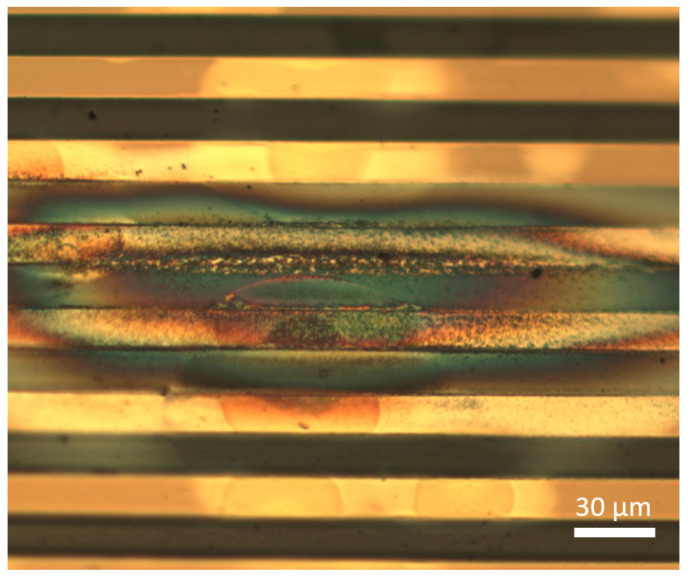
Gold conducting paths (30 µm pitch) of a flexible ECoG array embedded in two 5 µm thick polyimide layers. The array was used for long-term electrical stimulation tests in vitro in saline solution to investigate the electrode coating stability of PEDOT: PSS. Due to delamination between the polyimide layers and continuous electrical current pulses, gold dissolved and formed a thin gold layer, which led to short circuits between the conducting paths.

The research and development of flexible neural interfaces are of great interest not only for basic neuroscience, but also for applications in medicine, e.g., neural protheses [1,2]. Due to the softness of the brain tissue, also soft and flexible implantable materials are essential for reducing the foreign body response and, thus, enable chronic applications of the electrical interfaces [3]. These flexible materials need to be biocompatible to avoid any toxic reactions with the surrounding tissue. Currently, the most used materials for this purpose are the polymers parylene C, polyimide and SU-8 [4]. However, the negative photoresist SU-8 needs to be processed adequately to avoid cytotoxicity resulting from leachables [5]. Comparing the mechanical stability of parylene C and polyimide, the polymer polyimide has a superior stability, particularly for the application as a flexible substrate for neural implants [6]. However, due to the chemical inertness of polyimide after the complete curing process of the polyimide precursor, the adhesion to other polyimide layers or other materials is a challenge, which limits the reliability and long-term stability of polyimide-based neural implants. In a previously published work [7], we showed that after ca. 10 months in saline solution at 37 °C, delamination occurred between two polyimide layers, which increased the parasitic capacitance between adjacent conducting paths. This can lead to an increased signal crosstalk and, in the worst case, to a signal loss. To improve this electrical insulation stability of a polyimide/metal/polyimide sandwich layer in a saline environment, different methods, e.g., the chemical or oxygen plasma activation of the first polyimide layer surface, are used [8,9]. The current state-of-the-art method is still the use of a short oxygen reactive ion etching (RIE) treatment process to increase the polyimide to polyimide adhesion [10]. However, our current results show that, even when this oxygen plasma treatment is used, delamination between two polyimide layers can occur and even create short circuits, if the electrode array [11] is also used for the electrical neural stimulation; see Figure 1.

Nowadays, there are only a few methods published with the focus of investigating the long-term stability of flexible neural implants. One is the study of Takmakov et al., which uses accelerated aging of the neural implants with hydrogen peroxide at elevated temperatures [12]. This harsh treatment with reactive oxygen species is, however, incompatible with organic substrates such as polyimide. Another study by Rubehn et al. investigates the changes in the material properties of polyimide stored in saline solution [13]. Moreover, the water absorption performance of polyimide is well analyzed [14]. However, these studies do not represent the polyimide to polyimide interaction performance, which is crucial for the reliability of flexible neural interfaces. For this reason, we establish a test method, which uses interdigital electrode arrays embedded in two thin polyimide films. These test samples are stored in saline solution and a voltage between the electrodes is applied to assess the electrical insulation breakdown of the polyimide insulation. An additional ultrasonic treatment and elevated temperature accelerate the degradation process. Using the method presented in this article, differently treated samples with oxygen plasma are analyzed in terms of the long-term stability of the electrical insulation performance.

## 2. Materials and Methods

### 2.1. Design and Microfabrication Process of the Test Samples

**Figure 2:**
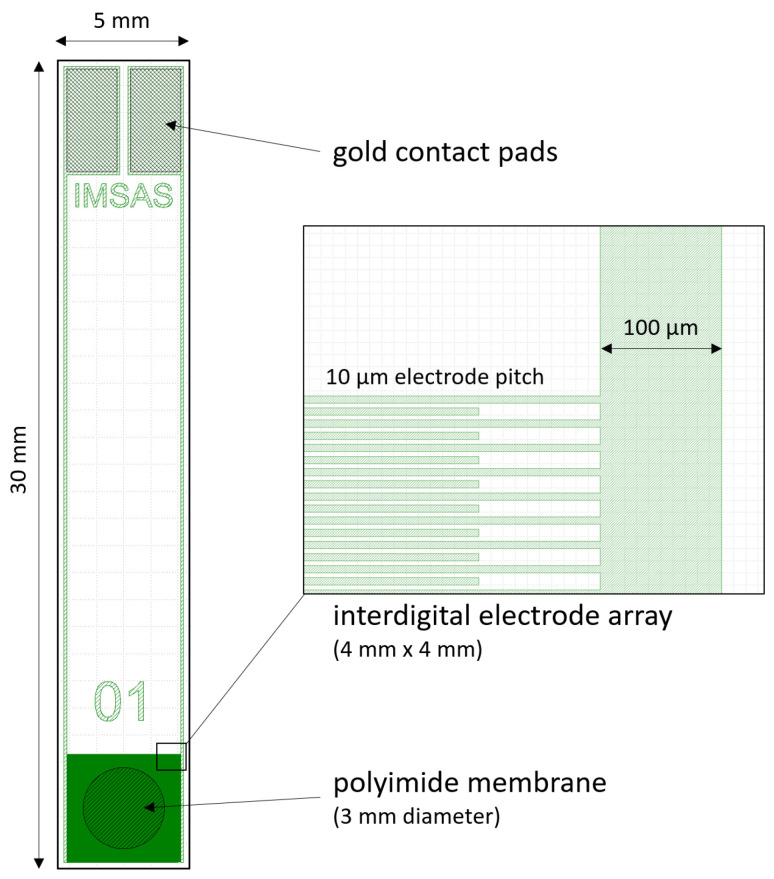
Design of the polyimide-based test sample. The enlarged cutout shows the interdigital gold electrode array with 5 µm wide electrodes and 5 µm spacing with a total area of 16 mm^2^. A 3 mm diameter polyimide membrane is located in the middle of the electrode array.

To investigate the long-term stability performance, test samples were designed and fabricated using microtechnology processes, which are also used for the fabrication of flexible neural implants [15]. The design of the test sample is shown in Figure 2. For the stability test of the polyimide electrical insulation, gold electrodes were arranged as interdigital structures embedded in two ca. 5 µm thin polyimide layers. Additionally, a 3 mm diameter membrane was opened to enable diffusion of saline solution from both sides and to allow mechanical stress to the polyimide/gold/polyimide sandwich structure. The used mask design, including three layers, can be downloaded from the Supplementary Materials. In total, 26 test samples were arranged on one wafer.

**Figure 3:**
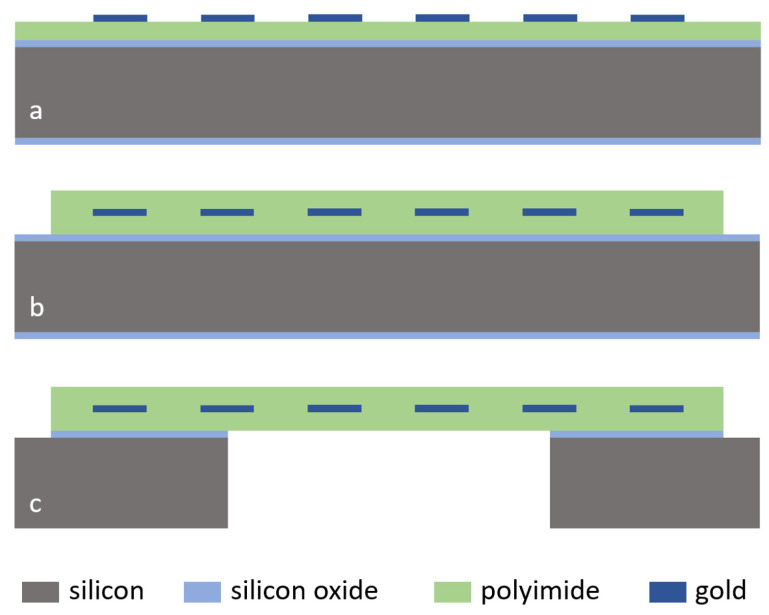
Microfabrication process flow: (**a**) thermal oxidation, polyimide coating and curing, gold deposition and structuring; (**b**) coating second polyimide layer after treatment of the first polyimide layer, structuring polyimide; (**c**) opening membrane using DRIE and oxide etching.

The microfabrication started with thermal oxidation of a 100 mm double-sided polished silicon wafer (380 µm thickness) to generate a 500 nm thick silicon oxide layer for electrical insulation to the substrate and an etch-stop layer during membrane opening, see Figure 3. For a strong adhesion between silicon oxide and polyimide, the adhesion promoter 3-Aminopropyltriethoxysilane (0.1 vol.%, Sigma-Aldrich Chemie GmbH, 82024 Taufkirchen, Germany) was applied. Directly afterwards, polyimide U-Varnish-S (UBE Europe GmbH, Germany) was spin-coated at 3000 rpm and cured using a stepped temperature profile up to 450 °C (5 °C/min temperature ramp) according to the product manual and a vacuum-hotplate (UniTemp GmbH, 85276 Pfaffenhofen/Ilm, Germany), resulting in a layer thickness of ca. 5 µm. A 300 nm thick gold layer was sputtered (100 W, DC magnetron) on polyimide and structured using the 1.8 µm thick positive photoresist AZ 1518 (MicroChemicals GmbH, 89079 Ulm, Germany) and the gold etching solution Au Etch 200 (NB Technologies GmbH, 28359 Bremen, Germany). The photoresist was afterwards stripped using AZ 100 Remover (MicroChemicals GmbH, 89079 Ulm, Germany). This structured gold layer was used for the interdigital electrode array, the conducting paths to the electrodes and the contact pads. Directly before coating of the second polyimide layer, the first polyimide layer was exposed to a pure O_2_ gas reactive ion etching process (up to 5 min) using an inductively coupled plasma tool (STS Multiplex ICP (Surface Technology Systems GmbH, 89073 Ulm, Germany), 13.56 MHz coil (800 W) and platen (25 and 50 W) generators). Only this polyimide treatment process was varied in this study to investigate the influence on the polyimide to polyimide long-term adhesion stability. Afterwards, the second polyimide layer was coated and cured the same way as the first layer. For opening the contact pads and removing polyimide in the wafer dicing area, a 20 µm thick AZ 9260 photoresist (MicroChemicals GmbH, 89079 Ulm, Germany) and an O_2_/CF_4_-reactive ion etching process were used. The photoresist was, afterwards, stripped again using AZ 100 Remover (MicroChemicals GmbH, 89079 Ulm, Germany).

For opening the polyimide membrane, a 10 µm thick AZ 9260 photoresist (MicroChemicals GmbH, 89079 Ulm, Germany) was applied on the wafer backside. The 500 nm thick oxide layer was etched in a CF_4_ RIE plasma. Afterwards, the 380 µm thick silicon layer was structured using a deep reactive ion etching process (DRIE) and a short O_2_ plasma was applied at the end to remove the passivation layer during DRIE. The photoresist was stripped in AZ 100 Remover. The silicon oxide etch-stop layer was removed in Buffered Oxide Etch BOE 7:1 solution (MicroChemicals GmbH, 89079 Ulm, Germany). After microfabrication, the test samples were separated using a wafer dicing tool without any additional protection of the polyimide layers during dicing.

### 2.2. Connector Assembly and Sample Setup

For the long-term test, the samples were stored individually in a tightly closed preparation glass with a rubber seal (15 mL volume, Th. Geyer Hamburg GmbH, 22419 Hamburg, Germany). A small 0.8 mm × 6 mm slit was milled into the plastic screw cap, through which the sample was passed. A 2-pin connector was glued onto the sample using the electrically conductive adhesive Elecolit 414 (Panacol-Elosol GmbH, 61449 Steinbach, Germany), which was cured at 70 °C for 20 min. Afterwards, the connector part of the sample and the cap slit were hermetically sealed with the two-component epoxy adhesive UHU PLUS ENDFEST (UHU GmbH & Co. KG, 77815 Bühl/Baden, Germany), which was also cured at 70 °C for ca. 2 h and left for a minimum of 24 h at room temperature before starting the long-term test.

### 2.3. Long-Term Test Method

**Figure 4:**
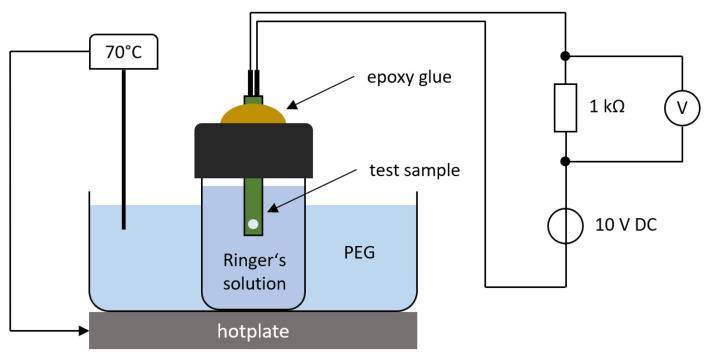
Test method setup with electrical circuit connection. The voltage over the 1 kΩ resistor was recorded and analyzed automatically with a National Instruments data acquisition card and a LabView software.

The test samples were stored in Ringer’s electrolyte solution (B. Braun Melsungen AG, 34212 Melsungen, Germany) at a constant temperature of 70 °C using a hotplate and temperature controller, see Figure 4. This elevated temperature accelerates the diffusion of saline into the polymer layers and speeds up the degradation process [16]. A DC voltage of 10 V was applied to the interdigital electrode array with a 1 kΩ resistor in series. The voltage over the resistor was monitored continuously using a National Instruments data acquisition card at a sampling frequency of 1 Hz. A LabView program analyzed the recorded data and automatically generated a time stamp when the voltage reached a threshold of 10 mV, which corresponded to an insulation leakage current of 10 µA. The current noise of the used measurement setup was measured around 1.3 µA; thus, only a break-down of the electrical insulation could generate this leakage current. To accelerate the aging process, a mechanical stress test was also applied to the samples using an ultrasonic (US) bath (35 kHz, 300 W power). This US test was performed weekly for 15 min starting one week after the insertion of the test samples into the saline solution. For this purpose, the glasses were taken out of the polyethylene glycol (PEG) solution and disconnected from the voltage source. The sonication was always performed at room temperature.

## 3. Results

### 3.1. Microfabrication Results of the Test Samples

**Figure 5:**
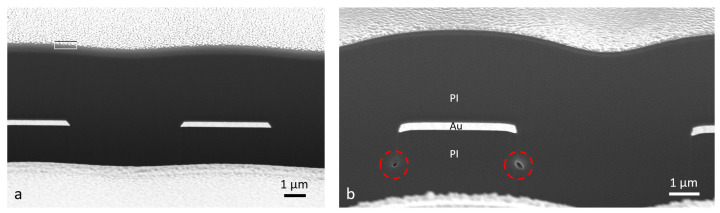
SEM images of the 5 µm polyimide/300 nm gold/5 µm polyimide cross-section: (**a**) 1 min oxygen RIE @ 25 W of the first polyimide layer; (**b**) 3 min oxygen RIE @ 25 W of the first polyimide layer. Voids are present at the PI/PI interface (red circles).

The presented microfabrication total process yield was ca. 80%. Most of the defects occurred in the photoresist during the photolithography of the gold layer, which resulted in a short circuit between the interdigital electrodes (10 µm pitch distance) after gold structuring. Only a few defects were observed in the polyimide layers. Figure 5 shows two SEM images of the polyimide/gold/polyimide cross-section created with a focused ion beam (FIB) cut inside the SEM vacuum chamber.

Due to the reactive ion etching of the first polyimide layer with pure oxygen plasma, a step in between the interdigital electrodes was created, which led to a wavelike surface topography of the second polyimide layer after coating and curing. Furthermore, several voids could be observed at the polyimide/polyimide interface for longer etching times over 2 min (see Figure 5b). This could lead to an aggregation of diffused water and accelerate the delamination process.

### 3.2. Connector Assembly and Sample Setup

**Figure 6:**
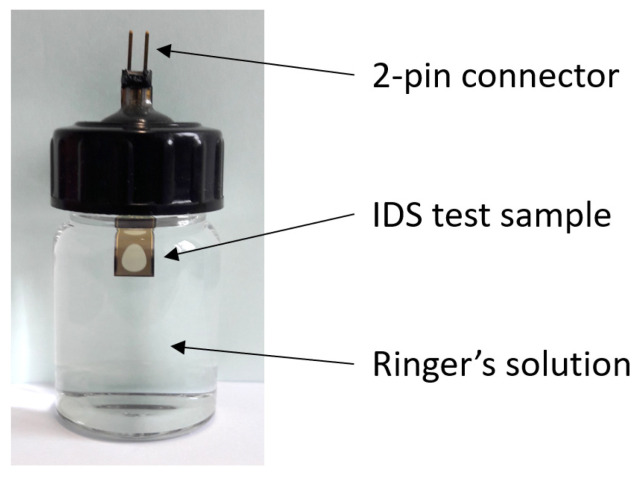
Test sample setup in Ringer’s electrolyte solution. The 2-pin connector was glued to the contact pads with an electrically conductive adhesive. Connector and slit in the plastic cap were hermetically sealed with an epoxy adhesive.

Figure 6 shows the final setup of the test sample stored hermetically sealed in a glass. The Ringer’s electrolyte solution was always filled directly before starting the long-term test. No evaporation of the electrolyte was observed during the total test period. The electrical connection of the two-pin connector to the sample using the electrically conductive glue and the encapsulation with the two-component epoxy adhesive achieved a yield of ca. 90%.

### 3.3. Long-Term Test Results

In total, 188 samples were tested using the presented method. An amount of 10 different oxygen RIE plasma surface treatments of the first polyimide layer was, therefore, investigated; see Table 1. For the bias power of 25 W and 50 W, a maximum etching time of 5 min and 3 min was defined, respectively.

**Figure 7:**
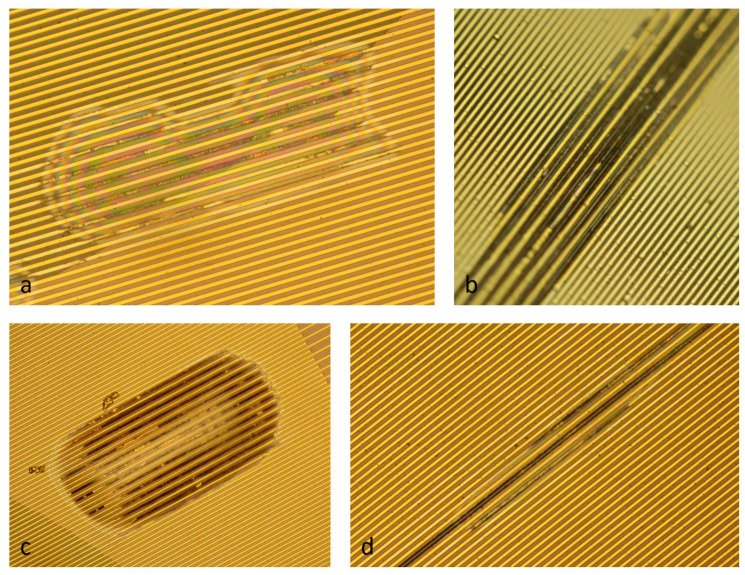
Microscope top view images of defect examples after the long-term test: (**a**) delamination occurred at the membrane transition and gold layer dissolved at anode electrode; (**b**) corrosion of gold layer on the PI membrane; (**c**) delamination and gold corrosion outside the membrane; (**d**) narrow delamination and gold corrosion.

The long-term stability was up to a maximum of 125 days. The defect types of the samples, which generated the measurable insulation leakage current, were comparable to the expected defect presented in the introduction. Some of these defect examples are shown in Figure 7. Due to delamination between the two polyimide layers, the electrical current could flow between the electrodes, which led to anodic corrosion and the dissolution of gold [17]. This simplified the optical localization of the defect origin after the long-term test.

**Figure 8:**
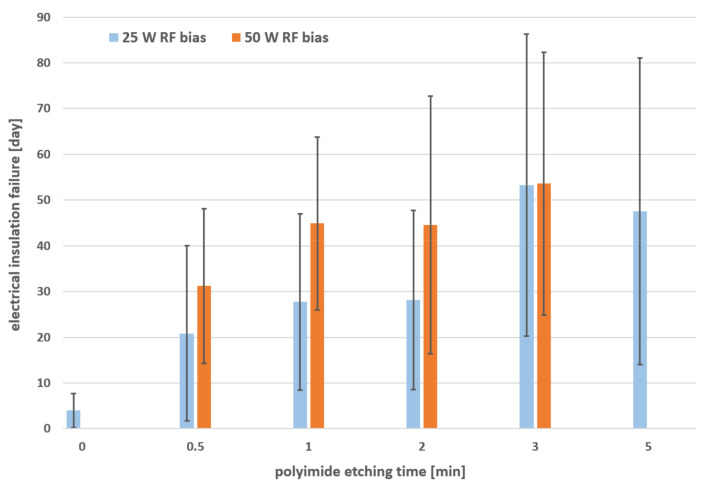
Long-term test results of 10 different polyimide surface treatments. Average values and first standard deviation (error bars) were plotted. The samples etched 0 min were not treated with O_2_ plasma for comparison.

For a direct comparison of the 10 different treatments, the long-term test results were plotted using the average values and the first standard deviation; see Figure 8. The results showed that the variation of the oxygen RIE plasma process (polyimide etching time and bias power) had a significant effect on the long-term stability of the samples. The samples which were not treated before the second polyimide coating (0 min) achieved only a marginal stability of 4.0 ± 3.8 days. This corresponded to the low adhesion strength between the two pristine polyimide layers [9]. Using the oxygen RIE plasma, the stability could be clearly increased; however, the deviation within the same plasma process was relatively large with up to 33.5 days (5 min at 25 W). Comparing the two different plasma treatments of 0.5 min at 25 W and 1 min at 50 W, the long-term stability could be increased from 20.9 ± 19.1 days to 44.9 ± 18.9 days, respectively. This corresponded to more than a doubled lifetime. By increasing the polyimide etching time, the average stability could be further increased; however, the deviation also increased.

## 4. Discussion

The polymer polyimide is often used as a flexible substrate for a variety of sensor applications due to its superior mechanical stability, electrical insulation and chemical inertness [18]. For neural implants, the electrical conducting paths composed of biocompatible and noble metals such as gold or platinum are embedded between two thin polyimide layers, which encapsulate the implant against the saline environment of the neural tissue [4]. For a long-term stable electrical insulation of the conducting paths, the adhesion between the two polyimide layers must be strong enough to prevent delamination, which is a challenge due to the chemical inertness of polyimide after it is completely polymerized [9]. This long-term stability is especially important for active neural implants, where electrical current is also applied for electrical stimulation of neural activity. Here, delamination could lead not only to an increased signal crosstalk during the recording of neural activity, but also to short circuits between conducting paths.

In a previously published paper, we assessed different surface treatments of polyimide to evaluate the adhesion strength between two polyimide layers [9]. As a result, the samples treated with oxygen reactive ion etching plasma were not peelable at all thus indicating the best approach to promote a strong adhesion between the PI layers. However, it was still unclear how the variation of the oxygen RIE plasma process affected the long-term stability in a saline environment. The presented long-term test method now gave the opportunity to evaluate this oxygen RIE plasma treatment.

The results of the long-term test showed that the variation of the oxygen RIE plasma process had a significant influence on the electrical insulation stability in saline solution. Without any treatment, it was also clear now that a chronic application was not possible (4.0 ± 3.8 days). The previously used process (0.5 min at 25 W bias) for the fabrication of neural implants at IMSAS cleanroom, which was, e.g., applied for chronically implanted flexible ECoG arrays [11] or intracortical probes [15], achieved a stability of 20.9 ± 19.1 days. The relatively high standard deviation showed that this short oxygen plasma process was not reliable and, thus, many of the fabricated implants could fail at early stages. By just doubling the plasma treatment time and bias power (1 min at 50 W bias), the long-term stability could be further improved to a value of 44.9 ± 18.9 days. Although the standard deviation remained almost the same, the average value could be more than doubled. Longer etching times could further increase the average values; however, the standard deviation clearly increased. This could be explained due to the presence of voids at the corners of the polyimide interfaces (shown in Figure 5b), which could accelerate the delamination process. For this reason, longer etching times (>2 min) were not preferable.

Although a step towards long-term stable neural implants was conducted in this study, the search for an ideal solution for the delamination problem between two polyimide layers is still ongoing. Another well-known approach is using additional intermediate layers, e.g., atomic-layer-deposited (ALD) Al_2_O_3_ as a diffusion barrier [19]; however, the adhesion between the different materials still remains a challenge.

## 5. Conclusions

In this study, a new method was presented for the evaluation of the long-term stability of the polyimide-based, flexible electrical insulation for chronic applications of neural implants, e.g., neural prostheses. Using this test method, we assessed different oxygen plasma treatments of the polyimide surface for increasing the adhesion strength between two polyimide layers. The results showed that the fine-tuning of this microfabrication process can significantly increase the lifetime of chronically implanted neural electrodes.

## Figures and Tables

**Table 1:** Overview of the number of test samples used for the analysis of the long-term stability.

Polyimide Surface Treatment	Total Test Samples
No treatment	19
0.5 min at 25 W bias power	22
1 min at 25 W bias power	18
2 min at 25 W bias power	15
3 min at 25 W bias power	17
5 min at 25 W bias power	19
0.5 min at 50 W bias power	20
1 min at 50 W bias power	17
2 min at 50 W bias power	22
3 min at 50 W bias power	19

## Supplementary Materials

The following is available online at https://www.mdpi.com/article/10.3390/mi12111279/s1, Lithography mask S1: IMSAS_IDS_PI_TEST.gds.
